# The health and health behaviours of Australian metropolitan nurses: an exploratory study

**DOI:** 10.1186/s12912-015-0091-9

**Published:** 2015-09-03

**Authors:** Lin Perry, Robyn Gallagher, Christine Duffield

**Affiliations:** Faculty of Health, University of Technology Sydney, Prince of Wales Hospital, East Wing Edmund Blackett Building, Randwick, 2031 NSW Australia; Charles Perkins Centre and Sydney Nursing School, University of Sydney, Sydney, Australia; Centre for Health Services Management, Faculty of Health, University of Technology Sydney, Sydney, Australia; Nursing and Health Services Management, Edith Cowan University, Mount Lawley, WA Australia

**Keywords:** Nurses, Health promotion, Workforce, Workplace, Health, Wellbeing, Lifestyle, Risk factors, Chronic disease, Sickness absence

## Abstract

**Background:**

Nurses make up the largest component of the health workforce and provide the majority of patient care. Most health education is delivered by nurses, who also serve as healthy living and behavioural role models. Anything that diminishes their health status can impact their credibility as role models, their availability and ability to deliver quality care, and is potentially disadvantageous for the health of the population.

Study aims were to investigate nurses’ overall health and the presence of chronic disease; to describe nurses’ health-related behaviours and to compare them to those of the general population, with both groups matched by age and gender.

**Methods:**

Cross-sectional descriptive paper-based survey of nurses from two Sydney metropolitan hospitals using established instruments and questions and measurements taken with standardised methods.

**Results:**

This nursing sample (*n* = 381) had a mean age of 39.9 (SD 11.7, range 20–67) years, Most (*n* = 315; 82.7 %) were female, worked full-time (80.0 %), and were shift workers (93.0 %). The majority (94.0 %) indicated good, very good or excellent health, despite 42.8 % indicating they had chronic disease. The most common risk factors for chronic disease were inadequate vegetable (92.6 %) and fruit intake (80.1 %), overweight and obesity (44.0 %) and risky alcohol intake (34.7 %); health screening behaviours were not ideal. Aside from overweight and obesity, these risk factors were more prevalent in nurses than the equivalent group of the New South Wales population, particularly for risky alcohol intake which was much more common in female nurses and most marked in those aged under 35 years. However, 80 % met the guidelines for physical activity, more than the equivalent group of the New South Wales population.

**Conclusion:**

There are early ‘warning signs’ concerning the health status of nurses. Despite perceiving current good health, support is required for nurses to prevent future chronic disease, particularly in the areas of nutrition and alcohol intake. With these concerns, the nursing workforce ageing and demands for care increasing, it is now time to implement health enhancing strategies for nurses.

## Background

Population ageing and increasing prevalence of chronic disease is seen across developed, low and middle income countries [1, 2]. Many of these chronic diseases are to some extent preventable and related to lifestyle and behavioural choices. Activities such as promotion of health literacy, health education and healthy living role modelling are therefore essential to reduce the burden of disease. People live with chronic diseases for many years, so provision of supportive care is also a health service priority. Nurses provide the bulk of both health education and health care; anything that diminishes their credibility as role models and health educators or their availability and ability to deliver quality care is potentially disadvantageous for the health of the population [3].

Demographic and workforce changes combine to make the health of nurses a priority for health service providers and policy makers. Global changes in population demographics are forecast to increase demand for healthcare services [4]. Chronic or non-communicable diseases associated with ageing are projected to increase by 15 % globally between 2010 and 2020. However, good long term health and disease prevention is possible through key healthy behaviours including limiting alcohol use, abstaining from tobacco, eating recommended amounts of fruit and vegetables, being physically active and maintaining a healthy weight [5]. Around 80 % of coronary heart disease and cerebrovascular disease have been estimated to accrue due to behavioural risk factors [4], so even small changes in behaviour have the potential to impact on health outcomes. Behaviour change activities need to be targeted as health behaviours differ between sexes and across age and geographical groups, with, for example, men more likely to have risky alcohol intake and low vegetable intake and women to have insufficient exercise, with obesity more prevalent in regional/ rural areas [6].

In addition to healthy lifestyles, the incidence and health impact of chronic disease can be minimised by regular screening and early intervention. For instance, regular cancer screening via mammography is recommended for women at risk and/or aged 50–69 years, biannual cervical testing for women who are sexually active/ aged 20–69 years and regular skin checks at individually determined frequency [7].

Long term, healthcare and prevention strategies are crucial to address these population trends and the World Health Organisation emphasises the need to strengthen the capacity of the health workforce to meet these demands [8]. Nurses provide the bulk of healthcare and are the main health education providers; highly visible, they are accessible role models for good health practices for their patients, families and the community. However, the nursing workforce faces the same health issues as the population it serves. On the other hand, nurses have advantages that should support their participation in health behaviours, including being well educated with high health literacy and the economic advantages of employment. Whether nurses’ health and health behaviours reflect this advantage is largely unknown.

In high income countries the nursing workforce is ageing [9] and to a greater extent than the populations from which they are drawn [10, 11]. Workforce shortages have become substantial and are predicted to continue and worsen [12]. One approach to managing this is to develop policies and strategies to slow the retirement losses that typically start to increase around age 50 and accelerate sharply at age 60 years [13, 14]. Nurses’ health is a crucial consideration for their retention in the workforce. Longer-serving nurses, particularly, are needed in the workforce for their experience and expertise, which is vital to support novice and less experienced nurses [15]. However, older nurses tend to have worse health and higher health-related productivity loss [16].

There is some evidence of risk to the health of nurses through obesity, lack of physical activity and poor diet, varying according to age and sex. The majority of nurses sampled did not meet recommendations for physical activity, fruit and vegetable or alcohol intake, with high levels of obesity [17, 18]. One study sampled nurses undertaking preregistration education [17], so the impact on their work capacity and ability to act as healthy role models was hampered from the beginning of their careers. Overall, however, little information is available about the health and health behaviours of the nursing workforce; this paper is part of the first stages of an initiative to address this gap.

The aims of this study were to indicate nurses’ overall health and the presence of chronic disease; to describe nurses’ health-related behaviours and compare these to those of the NSW general population, with both groups (nurses and the general population) stratified by age and gender.

## Methods

Survey design was used, employing a paper-based tool comprising previously validated instruments and questions. Physical measurements were sought of blood pressure, random capillary blood glucose, weight, height and waist circumference.

### The survey tool

This paper reports only the physical health-related components of the survey; mental health components are reported elsewhere [19]. Medical history, health screening (for cancers and indices of cardiovascular risk), preventive care and behavioural lifestyle-related characteristics were sought using questions from the Australian Longitudinal Study of Women’s Health [20] and the Western Australian Health in Men Study [21]. Items were chosen from these as both are population-based studies comprising validated questions. Demographic, social and occupational details were sought.

#### Physical health

Respondents’ views of their overall general health were sought using the relevant subscale of the Medical Outcomes Survey Short Form 36 (MOS SF-36) [22]. This questionnaire has been extensively tested for validity and reliability [23] and Australian reference values have been published following its use in the Australian Household, Income and Labour Dynamics in Australia (HILDA) Survey [24].

The presence of common chronic diseases was sought (e.g. hypertension, diabetes, depression), disease-related symptoms and current physical health-related medications.

Measurements were collected of height, weight, waist circumference, blood pressure and random capillary blood glucose. Body mass index (BMI) was calculated as weight (kg)/ height (m^2^); presence of overweight and obesity was identified by applying World Health Organisation criteria of overweight at BMI 25–29 kg/m^2^ and obesity at BMI ≥ 30 kg/m^2^ [25]. Criteria from the National Heart Foundation of Australia for risk associated with waist circumference were used: >80 cm for females and >94 cm for males [26]. Blood pressure thresholds were systolic blood pressure of ≥135 mmHg and diastolic blood pressure of ≥90 mmHg, or ≥130 mmHg systolic and ≥80 mmHg diastolic in the presence of diabetes [27]. Capillary blood glucose was considered elevated at ≥5.5 mmol/L, chosen as this is commonly accepted as the threshold for pre-diabetes and increasing risk of vascular disease has been indicated above this value [28]; serum cholesterol levels considered to pose risk for cardiovascular disease were those above the recommended threshold of 5.5 mmol/L [29].

#### Health related behaviours

Details of lifestyle-related factors included tobacco smoking, alcohol intake, physical activity, consumption of fruit and vegetables; fat intake was indicated by type of milk consumed (full, reduced or no fat). These data were classified for increased risk using methods from the 2009 NSW Health Population Survey [6]: of daily smoking, alcohol intake ≥5 drinks /day more than once per month [30], overweight/ obesity (BMI ≥25 kg/m^2^; [25]), insufficient physical activity with <150 min/week of moderate-vigorous activity [31]; risky saturated fat intake indicated by consumption of whole milk [32]; insufficient fruit and vegetable intake at <2 pieces of fruit/ day and <5 serves of vegetables/ day [33]. Each risk indicator or behaviour (excluding risky total cholesterol and capillary blood glucose levels due to small numbers) was allocated one point and summed to obtain a total potential cumulative risk score of nine points [6].

Participants were asked their participation and most recent timing of routinely available cancer screening: breast mammography, cervical smears, skin and bowel cancer checks. Responses were compared to current recommendations of mammography every two years for women aged between 50 and 69 years; bowel cancer screening for males and females aged 50 years and older; cervical screening every 2 years for women under the age of 69 years [7]. Skin checks were sought, according to whether this was conducted at least every 2 years.

### Sample

All Registered Nurses (RNs) and Enrolled Nurses (ENs; second tier of regulated nurse) employed on any form of contract at two study sites were invited to participate; a total of 1270 and 232 nurses, respectively. Both sites were acute tertiary referral hospitals in metropolitan Sydney, Australia.

### Data collection

Letters of invitation, Information Statements and copies of the survey were delivered and returned through the internal hospital post; reminders were delivered by email. Return of a completed survey was understood to convey consent to participate in this study. To obtain physical measurements of blood pressure, capillary blood glucose, weight, height and waist circumference, ‘measurement stations’ were set up on the wards over a 2-week period in each hospital and staff invited to attend and have their measurements taken by Registered Nurses using the hospitals’ Standard Operating Procedures (SOPs) for these activities. Alternatively, with the main points of the SOPs included at the back of the survey as a reminder, nurses could ask other nurses in their ward setting to collect these data. As conducting these measurements is part of the daily responsibility of hospital-based Registered Nurses no additional training was required. Data were collected over 4 months in 2011–2012 at the two sites.

### Data analyses

Data were entered and analysed using Statistical Package for the Social Sciences for Windows Version 22; were described using mean or median, standard deviation or percentiles, frequencies and percentages according to the level of the data. Comparisons were drawn between sample findings and those of age and sex matched samples from the NSW Health Population Survey 2009 [6] .

### Ethical considerations

All participants were supplied with written information about the project and had the opportunity to ask questions of a local research team member. With the approval of the Human Research Ethics Committees (HRECs), return of a completed survey was taken as consent to participate. Surveys were completed anonymously; no identifying data were collected. As all participants had a current nursing qualification it was possible that in completing the survey, a participant might recognise that particular responses indicated a degree of personal risk to health. It was assumed that any such participant would use their professional knowledge to take appropriate steps to seek clarification of survey findings and would, in the first instance, consult their General Practitioner. As survey respondents were not identifiable, the research team were not able to respond to any such finding. Ethical approval was granted from the South Eastern Sydney Local Health District and the University of Technology, Sydney HRECs (HREC refs 11/148 (LNR/11/POWH/242) and 2013000741).

## Results

Respondents (*n* = 381) had a mean age of 39.9 (SD 11.7, range 20–67) years; over one third (37.2 %) were 45 years of age and older (Table 1). Most (*n* = 315; 82.7 %) were female, worked full-time (80.0 %), and were shift workers (93.0 %); just over half were employed at Registered Nurse grade (56.7 %). These nurses were well-educated with most (*n* = 244, 64.4 %) having at least a Bachelor degree and 37.4 % (*n* = 143) with postgraduate qualifications.

**Table 1:** Socio-demographic and nursing characteristics

Characteristic (*n* = 381)	*N*	%^a^
Age group (years, *n* = 360)		
≤ 24	28	7.8
25–34	111	30.8
35–44	87	24.2
45–54	87	24.1
≥ 55	47	13.1
Country/ continent of birth (*n* = 372)		
Australia	172	45.7
United Kingdom and Ireland	68	18.2
Asia	65	17.4
Europe (non UK/ Ireland)	29	8.1
Other	34	10.2
Nursing experience (years) (*n* = 368)		
< 10	150	40.8
10–19	82	22.3
≥ 20	136	37.0
Work title		
Registered nurse	216	56.7
Enrolled nurse	18	4.7
Clinical nurse specialist	46	12.1
Clinical Nurse Educator/Nurse Educator	18	4.7
Clinical Nurse Consultant/Nurse Practitioner	38	10.0
Nurse Manager/Nursing Unit Manager	29	7.6
Manager/senior manager	12	3.1
Other	3	1.0
Highest qualification		
Bachelor degree	160	42.2
Bachelor degree plus certificate/diploma	84	22.2
Masters or doctorate degree	59	15.2
Certificate	41	10.8
Diploma	36	9

^a^Percentages may not total 100 due to missing values

### Nurses’ health

**Fig. 1:**
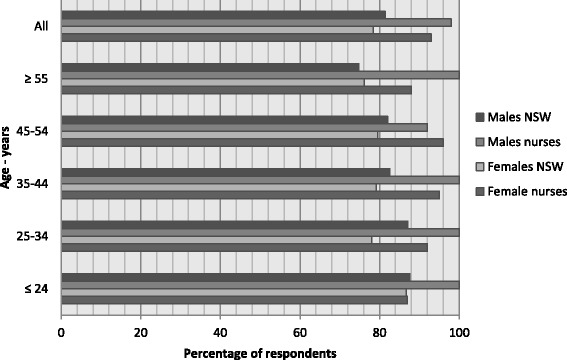
Self-rated health ≥ good (good, very good and excellent): New South Wales population versus nurses by sex and age (%) [6]

On a scale of 1 (poor) to 5 (excellent) nurses in the study rated their general health at mean 3.8 (SD 0.8), with 94.0 % (female 93.0 % and male 98.0 %) indicating good (*n* = 109, 28.5 %), very good (*n* = 174, 45.5 %) or excellent (*n* = 74, 19.4 %) health. All male and female nurse age groups except the youngest female age group (24 years or less) rated their health higher than the relevant age band of the NSW population (Fig. 1). While chronic diseases were present, they affected small proportions: *n* = 51 (13.4 %) reported psychiatric diagnoses, most commonly anxiety or depression (*n* = 49; 12.8 %); respiratory disease, predominately asthma, affected *n* = 48 (12.6 %); *n* = 37 (9.7 %) had been diagnosed with a cancer; some form of arthritis affected *n* = 29 (7.6 %); diabetes, pre-diabetes or gestational diabetes had been diagnosed for *n* = 29 (7.6 %) and cardiovascular disease and stroke affected *n* = 55 (14.4 %), *n* = 46 (12.1 %) of whom listed hypertension. Altogether *n* = 163 respondents (42.8 %) identified between one and six chronic disease diagnoses each.

The mean (SD) of *n* = 275 measured systolic (116.7 (14.6) mmHg) and diastolic (71.5 (11.3) mmHg) blood pressure readings were within normal ranges; a small proportion had an elevated systolic (≥135 mmHg or ≥130 mmHg in the presence of diabetes; *n* = 26, 9.4 %) or diastolic (≥90 mmHg or ≥80 mmHg in the presence of diabetes; *n* = 17, 6.2 %) measurements, indicating the need for further assessment. Random capillary blood glucose levels were tested (*n* = 140); with *n* = 17 (4.5 %) already having a diabetes diagnosis, the mean (SD) values were 5.2 (1.4) mmol/L, with *n* = 50 (35.7 %) at ≥5.5 mmol/L, conferring increased risk of developing diabetes and vascular disease. For *n* = 106 respondents cholesterol values were self-reported at mean (SD) 4.8 (1.4) mmol/L, with *n* = 25 (23.6 %) reporting levels greater than the recommended threshold of 5.5 mmol/L.

Nurses were asked if and how frequently they experienced potential disease symptoms of respiratory or cardiac origin, back and joint pain/ stiffness, urinary, bowel and oro-dental problems and whether they sought help for these (Table 2). Only 66 respondents (17.3 %) were free of these eight groups of symptoms, and overall respondents experienced symptoms in a median of two categories (25, 75 quartile scores 1–3 categories). Asked how much bodily pain they had experienced in the last 4 weeks, *n* = 101 (26.5 %) reported no pain whilst *n* = 59 (15.5 %) had experienced moderate and *n* = 20 (5.2 %) severe/ very severe pain.

**Table 2:** Disease related symptoms experienced sometimes/ often and help seeking (n, %)

Symptom type	Experienced sometimes		Experienced often		Sought help	
	*n*	%	*n*	%	*n*	%
Breathing difficulties	19	5.0	3	0.8	15	3.9
Chest pain	8	2.1	2	0.5	7	1.8
Palpitations	31	8.1	4	1.0	2	0.5
Back pain	108	28.3	45	11.8	38	10.0
Stiff joints	78	20.5	39	10.2	36	9.4
Urinary problems	19	5.0	7	1.8	16	4.2
Bowel problems	31	8.1	17	4.5	18	4.7
Oro-dental problems	42	11.0	11	2.9	35	9.2

### Health risk indicators

**Fig. 2:**
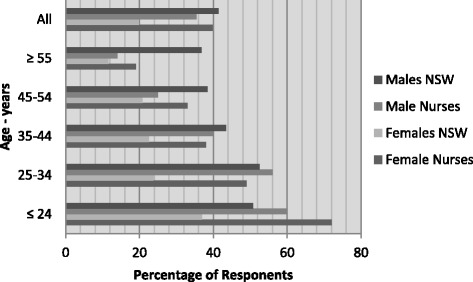
Risky alcohol intake (≥5 drinks/day more than once per month (NHMRC 2009)): New South Wales population versus nurses by sex and age (%) [6]

Risky alcohol intake (≥5 drinks/day more than once per month) was reported by *n* = 123 respondents (34.7 %; 39.7 % of females and 35.4 % of males). Female nurses were more likely to report risky drinking habits than the equivalent group of the NSW population; evident at all age bands, this was most marked in those aged under 35 years (Fig. 2). For males a higher proportion with risky alcohol intake only occurred in nurses aged 34 years and younger.

**Fig. 3:**
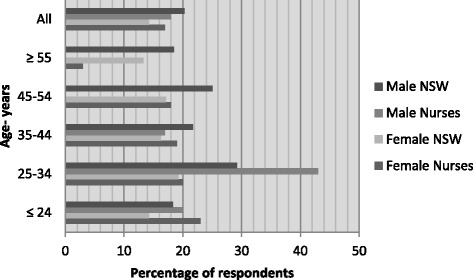
Daily smokers: New South Wales population versus nurses by sex and age (%) [6]

With *n* = 66 (18 %) self-reporting as current smokers, *n* = 25 respondents (6.8 %) described themselves as daily smokers; this comprised 17.7 % of female and 19.7 % of male nurses, although none of the males aged 45 years or more reported current smoking. Female nurses were more often current smokers than the equivalent age groups of the NSW population except in the oldest age category (Fig. 3).

**Fig. 4:**
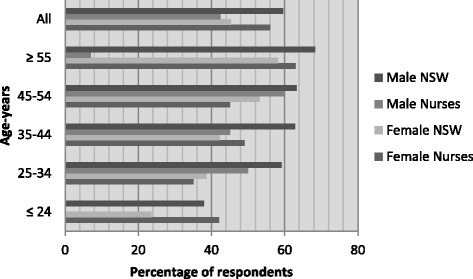
Overweight and/or obese: New South Wales population versus nurses by sex and age (%) [6]

With weights and heights available for *n* = 293, respondents had mean (SD) BMI of 25.4 (5.3) kg/m^2^ for female and 25.1 (5.7) kg/m^2^ for male nurses. One hundred and twenty nine (44.0 %) were classified as overweight or obese (43.8 % of female and 44.2 % of male nurses). For males of all age categories this was less than the NSW population, but the picture was more mixed for female nurses. A general trend to increasing BMI with age was clear, except for the small group of youngest age female nurse respondents (Fig. 4).

Waist measurements were available for *n* = 260. Increased cardiovascular risk represented by central distribution of adiposity (measurements >80 cm for females and >94 cm for males) was present in 56.9 % of female (*n* = 124) and 19.0 % (*n* = 8) of male nurses, fairly consistently distributed across the ages from 17.6 % of the youngest group, to 50.6 %, 56.7 %, 50.9 % and to 25.0 % of those aged 55 years and over.

### Healthy choices and behaviours

**Fig. 5:**
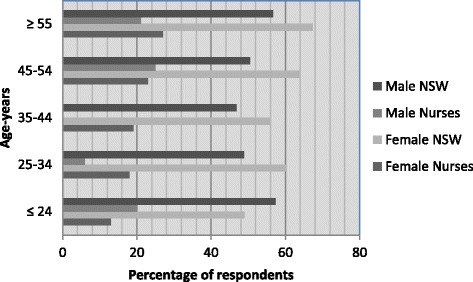
Fruit intake two or more pieces/day: New South Wales population versus nurses by sex and age (%) [6]

**Fig. 6:**
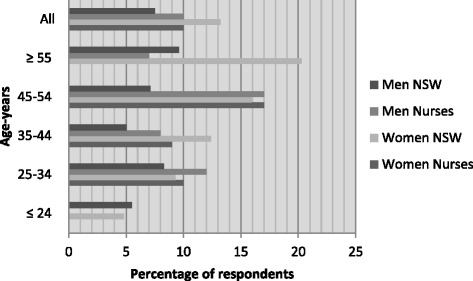
Vegetable intake five or more serves/day: New South Wales population versus nurses by sex and age (%) [6]

Less than one in five nurses (18.1 %) had fruit intake that met guideline recommendations for health of two or more pieces per day, with similar proportions for male (*n* = 8; 21.2 %) and female (*n* = 60; 27.0 %) nurses. All age groups of both genders of nurses had markedly smaller proportions of healthy fruit eaters than the NSW population (Fig. 5). However, even fewer nurses met the recommended vegetable intake of five or more serves of vegetables per day (*n* = 36; 9.4 %), regardless of gender at *n* = 1 (1.5 %) of male and *n* = 29 (9.2 %) female nurses. No-one of either gender in the youngest age group (up to 24 years of age) and no oldest age female nurses (55 years or over) met recommendations. Altogether, only 25 respondents (6.6 %) met both fruit and vegetable intake recommendations. Nurses’ vegetable consumption was often markedly worse than age and sex-matched state population groups (Fig. 6).

Use of reduced/ low fat rather than full fat milk has been accepted as an indicator of managing saturated fat intake; a minority of nurses (*n* = 93, 25.1 %) consumed full fat milk with most (*n* = 255, 67.2 %; males *n* = 44, 75.8 %; females *n* = 217, 74 %) of those who reported consuming milk across all ages consuming reduced/ skimmed fat, soy or other low fat milk. This is in contrast to the high proportion of the NSW population reporting drinking full fat milk: males 51.1 %, females 40.5 %. The pattern of cheese consumption was more mixed: with 65 respondents (17.6 %) not consuming any cheese, *n* = 49 (13.2 %) ate multiple different types; hard/ firm (more usually full-fat) cheese was consumed by *n* = 142 nurses (37.3 %) whilst *n* = 114 (30.9 %) more often preferred lower fat soft, ricotta, cottage or cream cheese. The distribution of responses across the sexes was similar. Choice of spreads was similarly diverse with 98 respondents (26.9 %) using none, *n* = 128 (35.2 %) used butter or butter and margarine blends, *n* = 83 (22.8 %) preferred mono or polyunsaturated spreads. Again the distribution across the sexes was similar. Finally, whilst *n* = 19 (5.1 %) respondents did not eat bread, nearly double this number (*n* = 35, 9.5 %) consumed multiple types. In total *n* = 73 (19.8 %) respondents chose white or white high fibre bread, whilst *n* = 132 (65.6 %) consumed wholemeal, multigrain or rye bread.

**Fig. 7:**
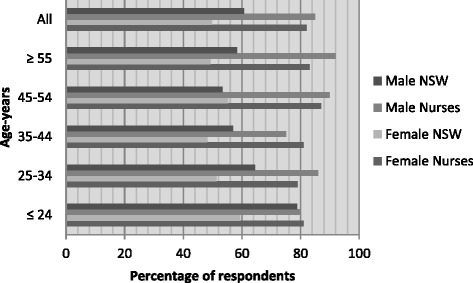
Meets physical activity recommendations (≥150 mins/week of moderate/vigorous exercise): New South Wales population versus nurses by sex and age (%) [6]

Physical activity participation rates were at a high level. The majority (*n* = 273, 82 %) met the Australian physical activity recommendations for health of ≥150 min per week (female 81.7 %, male nurses 85.2 %). Across almost all age groups and both genders, nurses exceeded the NSW population proportions substantially (by 20 % or more) except for the youngest group of male nurses (Fig. 7).

Overall, there were no significant differences between the health behaviours and risks of male compared to female nurses, although with only 66 male respondents this may reflect Type 1 error. Summing the nine health risks for chronic disease described in the NSW Health Population survey [6] (Table 3), of *n* = 231 respondents with complete data, *n* = 158 (68 %) demonstrated being ‘at risk’ in a mean of 3.9 categories (SD 1.3, range 0–7).

**Table 3:** Risk factors for chronic disease for the Australian population [6]

Risk factor (*n* = 342)	*N*	%^a^
Daily smoking	25	7.3
Risky alcohol intake (≥5 drinks/day >1/month)	123	34.7
Overweight/obese (BMI > 25 kg/m^2^)	129	44.0
Current high blood pressure (systolic blood pressure of ≥ 135 mmHg or ≥ 130 mmHg in the presence of diabetes or diastolic blood pressure of ≥ 90 mmHg or ≥ 80 mmHg in the presence of diabetes)	26	9.5
Waist circumference at risk (>80 cm for females and > 94 cm for males)	132	50.9
Physical activity insufficient (<150 min/week of moderate/vigorous activity)	69	20.0
Saturated fat intake risky (consumption of full fat milk)	93	25.1
Fruit intake insufficient (<2 or more pieces/day)	274	80.1
Vegetable intake insufficient (<5 or more serves/day)	317	92.6
Cumulative risk score (potential 0–9), mean, SD	3.87	1.35
Median 4, range 0–7, 25 % 3, 75 % 5		

^a^percentages vary to missing values

### Nurses’ patterns of behaviour

Sample size made it difficult to detect significant behavioural patterns; for example, although the average time spent in moderate-vigorous exercise including chores in the previous week totalled 7.8 h versus 10.5 h for overweight/ obese versus healthy weight nurses, this was not significantly different (t = 1.704, *p* < 0.089). However, suboptimal behavioural responses to health risks were indicated. For example, hypertension is an important modifiable risk factor for cardio-vascular disease, and effective therapies are readily available. However, of *n* = 46 (12.1 %) recording a diagnosis of hypertension, only *n* = 26 (56.5 %) reported anti-hypertensive medication and *n* = 17 (37 %) supplied blood pressure values above normal ranges [27].

### Nurses’ health screening and monitoring

Nurses’ attention to health screening and monitoring was generally suboptimal although some checks were better completed than others. Within the previous year *n* = 315 respondents (82.7 %) reported checking their blood pressure and *n* = 220 (57.7 %) their blood glucose; *n* = 189 (49.6 %) reported their total cholesterol level and *n* = 131 (34.4 %) had their skin checked. Of female nurses, *n* = 136 (45.6 %) reported a cervical smear within the last two years and *n* = 74 (54.4 %) had experienced abnormal findings at some point in their lives; *n* = 29 (9.7 %) had never had one or didn’t know when the last one occurred. Of the 76 female nurses aged 50 years and older, *n* = 24 (31.6 %) reported mammography within the previous 2 years, with *n* = 35 of the *n* = 140 (25.0 %) female nurses aged 40 years and older reporting mammography within the previous 2 years; *n* = 29 (20.7 %) had experienced abnormal findings at some point. Altogether, 48 of the 78 respondents aged 50 years and older reported any bowel screening, *n* = 16 (20.5 %) within the previous 2 years; *n* = 10 (12.8 %) reported abnormal findings.

## Discussion

The strength of this study lies with its unique insights into the health profile of the nursing workforce in the Australian public health sector. In summary, findings revealed that these nurses generally self-rated their health as good-very good, and higher than comparable samples from the NSW population. However, there were indicators that nurses were facing important health issues with more than 40 % reporting at least one chronic disease, and few free of symptoms of chronic ill health. These aspects may impact on current health promotion roles and work capacity but a greater concern is the implications for the future of the prevalence of risk factors for chronic disease.

Measured parameters indicated a degree of health risk: approximately 10 % had elevated blood pressure; blood glucose levels indicated more than one third at increased risk of developing diabetes and vascular disease; cholesterol values were elevated in almost one quarter. Overweight or obesity affected more than 40 % of both female and male nurses and almost half were at increased cardiovascular risk from central adiposity. The prevalence of many of these risk factors was similar or greater than the local population, indicating the strength of the influence of environment and societal factors on health behaviours despite health literacy and experience with the impact of risk factors on chronic disease. Few other studies have compared nurses’ health to that of the general population. In Australia Bogossian et al. [34] found similarly mixed comparisons, with many age bands of nurses with greater overweight/ obesity than comparable groups of the population in general. An Alabama study noted worse health related quality of life and overweight/ obesity for nurses, but findings may have been affected by poor response rate [35].

In three health behaviour areas, risky alcohol intake, fruit and vegetable intake and current smoking, nurses were at much higher risk than the general population, indicating areas where further study and intervention could be beneficial. More than one third of nurses indulged in risky alcohol intake, which occurred across the board but was more marked in younger female and male nurses. These were substantially higher risky alcohol intakes than reported in another study of Australian and New Zealand nurses and midwives which found that 13.9 % engaged in harmful daily drinking; comparisons were hampered by differing definitions (their threshold was >2 drinks/day) [36]. This drinking pattern was increased in nurses who worked longer hours (Odds Ratio (OR) 1.26 in those working >40 h/week and OR 1.46 in those working >50 h/week after adjusting for other important factors). Of particular concern is the alcohol intake of younger people.

Physical activity participation rates were high with more than 80 % meeting recommendations. These high rates of physical activity are commendable. Further investigation is warranted to identify patterns of physical activity as another Australian study identified the key role of leisure time physical activity in relation to decreasing sick leave [37].

Nurses’ health monitoring and screening generally was less than desirable. Whilst more than 80 % checked their blood pressure and around half their blood glucose (not hard for clinical nurses to do) and cholesterol, cancer screening was less adequate. Where this occurred, a high proportion reported abnormal findings; for example, only one third had their skin checked, and one third of these reported abnormalities. Under half had a cervical smear in line with recommendations, and half reported abnormal findings. Less than one third reported mammography in line with Australian recommendations; one fifth reported abnormal findings. One in five had undergone bowel screening, with one in eight reporting abnormalities. Given that screening programmes are required to support net health benefit [7] there is clearly potential for health gain if such screening rates can be increased.

In the face of increasing demands for healthcare and predicted workforce shortfalls, nurses’ ability to provide services may be limited by their health status, as will their capacity to act in health promotion roles. This group of nurses rated their health status similarly to that of the equivalent general population, but nurses tend to have higher BMI and pain scores and an increased likelihood of chronic health problems [16, 35]. For the future, nurses whose health status is already compromised in their ‘younger’ years, as found in this and another study [17], may face increased risk of adverse effects as they continue employment in the profession.

These findings contain potential ‘warning signs’ for workforce planning. Warnings derived from modelling demographic data have been sounded for at least 15 years [9]. This study begins the process of detailing what such warnings entail for working nurses and their employers. Nurses aged 45 years and above have been considered ‘older’ [38]; more than one third (37 %) of this sample were in this age bracket. Many countries, including Australia, have policies to encourage older people to stay in work as workforce shortages persist across many industries, particularly nursing [11]. Evidence from many countries indicates that a large proportion of RNs remain in employment beyond the age of 55 years [11, 14]. Employers should consider how workplace conditions may be modelled to promote retention of older nurses, many of whom may be challenged by their level of health. This might include, for example, offering opportunities for staff to move from fast-paced to less stressful work settings if they prefer [14]; offering flexible shift systems and flexible rostering, and greater access to part time working without loss of grade [10, 39].

Workplace health initiatives also offer opportunities, such as provision of healthy foods, facilities to heat and eat own food, amenities to support cycling or walking to work, exercise sessions etc. The workplace has been described as an advantageous site for health promotion, offering a captive audience for a large part of the week and the support of peers and colleagues [40, 41]. Peer support can have both a positive and negative effect as peers can be equally engaged in poor health or risky health behaviours. However, this has been little explored for nurses [42]. Nurses’ own experiences in relation to healthy lifestyles may significantly impact how they engage with health promotion for their patients [43] but there is currently little understanding of how nurses’ attitudes to risk factor reduction in their patients may be affected if they themselves struggle or, worse, don’t engage in healthy behaviours. Perhaps implementing and focussing on healthy changes in nurses’ own lives will ensure nurses are better able to participate in work and ultimately are more effective role models [44].

This study has some limitations. Some differences in demography from the two hospitals were evident when compared to the NSW and Australian nursing population. The mean age of these respondents was 39.9 years; younger than the average age of NSW nurses (43 years) [45]; fewer were aged 50 years and older than the Australian population of nurses (24.6 % compared to 36.3 %) [46]. Study findings may not be representative of the NSW or Australian nursing population and further work with larger / more representative samples is required. Most data were self-report, and the potential for bias in this form of data is well-recognised. Physical measurements were collected; whilst no formal inter-rater reliability checks were undertaken, measurements were made by staff with daily responsibility for such measurements from patients.

## Conclusion

It is important for nurses to be healthy, not only for themselves but also for the population which requires their services. Results from this study indicate that while nurses rated their health as little different from that of the general population, there were indications of common risk factors for chronic disease, some to a greater degree than in the population. It is now time to consider implementing health enhancing strategies, as demands for care increase and the nursing workforce ages. Health behaviours are not solely an individual responsibility. Policy-makers and managers in nursing and healthcare need to play their parts in making the workplace a positive environment for healthy working, supportive of nurses in health promotion and enabling them to role model what they teach.

## Abbreviations

BMI: Body mass index
EN: Enrolled nurse
HILDA: Household, income and labour dynamics in Australia Survey
MOS SF-36: Medical outcomes survey short form 36
NSW: New South Wales
OR: Odds ratio
RN: Registered nurse
SD: Standard deviation
UK: United Kingdom
